# Effectiveness of metformin pretreatment for stroke severity: A propensity score matching study

**DOI:** 10.1111/cns.70004

**Published:** 2024-08-21

**Authors:** Bowei Zhang, Scott Silverman, Lee H. Schwammn, Xunming Ji, Aneesh B. Singhal

**Affiliations:** ^1^ Harvard Medical School Boston Massachusetts USA; ^2^ Department of Neurology Massachusetts General Hospital Boston Massachusetts USA; ^3^ Department of Neurology Xuanwu Hospital of Capital Medical University Beijing China; ^4^ Department of Neurology Yale School of Medicine New Haven Connecticut USA; ^5^ Department of Neurosurgery Xuanwu Hospital of Capital Medical University Beijing China

**Keywords:** acute stroke therapy, cerebral infarction, neuroprotection, prevention, stroke, treatment

## Abstract

**Background and Objective:**

Metformin pretreatment might have neuroprotective effects. We aimed to determine the therapeutic effects of the antidiabetic medication metformin on ischemic stroke severity and discharge outcomes.

**Methods:**

We analyzed data on 1303 ischemic stroke patients who were on antidiabetic medications from the Massachusetts General Hospital (MGH) Advanced Comprehensive Stroke Center dataset (*n* = 8943, 2012–2022). We applied propensity score matching (PSM) and inverse probability of treatment weighting (IPTW) analyses to investigate the effect of current usage of metformin (versus alternate antidiabetic treatment) on acute stroke clinical severity and discharge outcomes.

**Results:**

Of the 1303 patients who were on antidiabetic medications at the time of stroke admission, 730 (56%) were taking metformin. Metformin users were younger and more frequently had hypertension, whereas less frequently had prior CAD, AFib, and chronic kidney disease. The clinical features and laboratory values of the two groups were evenly distributed after PSM. Metformin‐treated patients had statistically significant lower stroke severity on admission [National Institutes of Health Stroke Scale (NIHSS) (median, interquartile range) 3.0 (1.0–8.0) vs. 4.0 (2.0–11.3), *p* = 0.011], better functional independence at discharge (modified Rankin scale score 0–2, 36.3% vs. 25.4%, *p* < 0.001) and less in‐hospital mortality (4.5% vs. 11.3%, *p* = 0.018). IPTW analysis results were consistent with PSM results.

**Conclusions:**

Among diabetic patients with acute ischemic stroke, metformin appears to confer neuroprotection. Our results extend previous findings to the general stroke population. Stroke patients with diabetes mellitus who were treated with metformin prior to stroke, even when combined with additional antidiabetic medications, experienced less severe strokes upon admission and had better functional outcomes during hospitalization.

## Graphical abstract

This propensity score matching analysis of our stroke dataset found that diabetics being treated with metformin had milder clinical stroke severity, less in‐hospital mortality, and better functional outcomes compared to those who were treated with alternative antidiabetic medications at the time of their ischemic stroke. Among diabetic patients with acute ischemic stroke, metformin appears to confer neuroprotection.

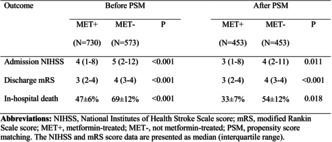

## 1 INTRODUCTION

Stroke is a leading cause of disability and death. Diabetes is a well‐established risk factor for stroke, conferring more than twice the risk of ischemic stroke.^1^ Patients with diabetes who experience a stroke generally face a higher risk of disease severity, an increased rate of complications, including hemorrhagic transformation, and poorer outcomes.^2^, ^3^ Therefore, investigating stroke patients with diabetes is of great significance in stroke management.

Metformin, an oral antidiabetic medication belonging to the biguanide class, is a common primary treatment for type 2 diabetes. It has shown neuroprotective effects in animal stroke models by improving insulin sensitivity, reducing inflammation, and lowering oxidative stress via the activation of adenosine monophosphate‐activated protein kinase (AMPK), an enzyme involved in cellular energy regulation.^4^ Prior studies have shown that stroke patients with diabetes have lower case fatality and disability rates if they are on metformin.^5^ Additionally, stroke patients receiving intravenous thrombolysis also experience less severe strokes if they are on metformin.^6^

This study aimed to investigate the effects of metformin on ischemic stroke clinical severity, in‐hospital mortality, and discharge functional outcome in our large cohort of consecutive stroke admissions.

## 2 METHODS

### 2.1 Study design and study population

We retrospectively analyzed the Massachusetts General Hospital Advanced Comprehensive Stroke Center dataset, 2012–2022. The study population comprised ischemic stroke patients with a prior history of diabetes mellitus who were on at least one antidiabetic medication at the time of stroke admission.

### 2.2 Data variables

Admission variables included patient demographics (age, sex, race); past medical history (hypertension (HTN), dyslipidemia, coronary artery disease (CAD), atrial fibrillation (AFib), chronic kidney disease (CKD), stroke, and smoking; medications (antiplatelet agents, anticoagulants, statins, antihypertensives, antidiabetic medications); and clinical and laboratory measurements (blood pressure, glucose level, hemoglobin A1C (HbA1C), serum creatinine, international normalized ratio (INR), National Institutes of Health Stroke Scale (NIHSS) score). Discharge variables included the modified Rankin Scale (mRS) at discharge and in‐hospital mortality.

### 2.3 Outcome

The primary outcome was a comparison of stroke clinical severity, measured by NIHSS, between metformin users versus nonusers. Secondary outcomes included a comparison of the mRS at discharge, functional independence (mRS 0–2) at discharge, and in‐hospital mortality.

### 2.4 Statistical analysis

#### 2.4.1 Descriptive analysis

Continuous variables are presented as mean ± standard deviation (SD) for data with normal distribution and median (interquartile range) for data with non‐normal distribution as assessed by the Kolmogorov–Smirnov test or histogram. Categorical variables are presented as numbers (percentage). To evaluate the balance of the baseline characteristics before and after PSM, the absolute standardized mean difference (SMD) was used for continuous variables and the absolute mean difference for categorical variables.

#### 2.4.2 Missing data

Patients with missing values for any outcome measure or metformin status were excluded from the analysis. Other missing values were handled using multiple imputations based on the assumption of missing at random. A total of 100‐fold multiple imputations were applied to the dataset with missing values (*m* = 100) to improve precision. Missing values were low‐density lipoprotein (LDL) level (8.6%), HbA1c (10.3%), glucose level (4.3%), serum creatinine (1.3%), INR (5.4%), and blood pressure (1.7%).

#### 2.4.3 PSM and IPTW

Propensity scores were calculated to address the issue of nonrandom assignment of treatment. The propensity score was estimated for each patient using a logistic regression model that included baseline variables as covariates, with the metformin‐user (MET+) group as the dependent variable. PSM variables were selected based on previous studies and investigators' judgment. The matching ratio was 1:1. The quality of matching was assessed by standardized differences of the mean (SMD) of the covariates before and after matching. Covariates with SMD <0.1 were considered balanced.

After PSM, stroke clinical severity (i.e., the median NIHSS score) was compared between the MET+ and MET− groups using the independent samples Wilcoxon test. Secondary outcomes, including the mean discharge mRS score, functional independence (mRS scores 0–2), and in‐hospital mortality, were compared using Student's t and Chi‐square or Fisher's exact tests, as appropriate.

We used IPTW analysis in addition to PSM to further adjust for confounding and test the consistency of our hypothesis.^2^ Propensity scores were utilized to calculate the IPTW. This approach allows for balancing the distribution of baseline covariates between treatment groups, which can lead to a more accurate estimation of treatment effects.

Finally, we investigated the treatment effect size for primary outcome across the following subgroups: age (<75 years vs. ≥75 years), sex (male vs. female), CAD (yes vs. no), HTN (yes vs. no), AFib (yes vs. no), previous stroke (yes vs. no), CKD (yes vs. no), HbA1c (<7.0% vs. ≥7.0%), and admission glucose (<140.0 mg/dL vs. ≥140.0 mg/dL).

Data analyses were conducted using Stata IC/16.1 (Stata Corp., College Station, TX) and R (Version 2022.12.0 + 353). R packages Amelia, mice, MatchIt, VIM, cobalt, WeightIt, and geepack were used. Statistical significance was determined using a two‐tailed *α* level of 0.05. The study was reported according to STROBE (Strengthening the Reporting of Observational Studies in Epidemiology).^3^

#### 2.4.4 Standard protocol approvals, registrations, and patient consents

The study was reviewed and approved by the Mass General Brigham Institutional Review Board. Informed consent was waived given retrospective database analysis and chart review design.

#### 2.4.5 Data access and availability statement

A.S. and B.Z. have full access to the data used in the analyses in the article. Data used for analysis are presented in the tables and figure in this article. Deidentified data will be shared after ethics approval if requested by other investigators for purposes of replicating the results.

## 3 RESULTS

### 3.1 Baseline characteristics

**FIGURE 1:**
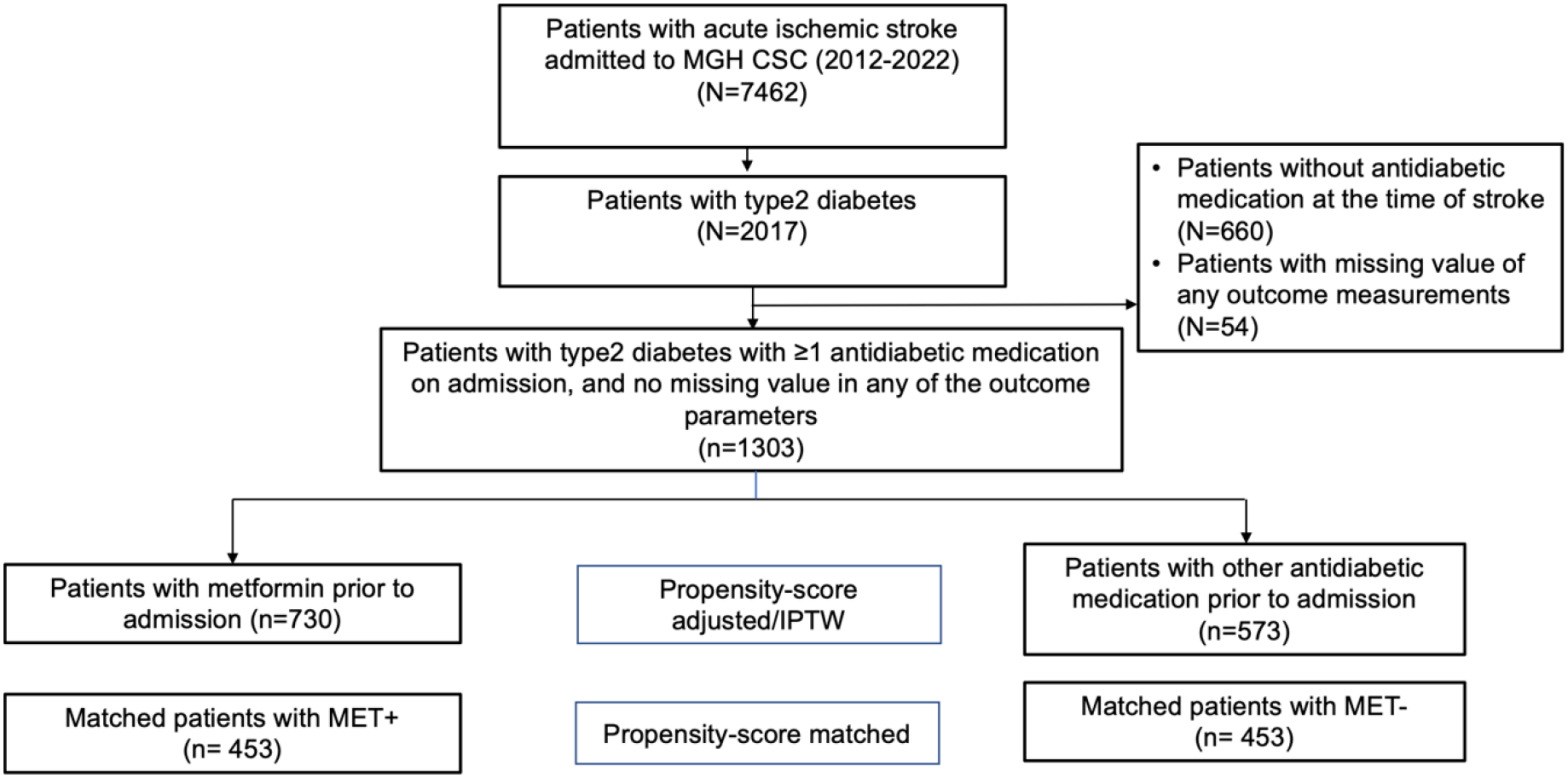
Flowchart of the patients' inclusion in this study. CSC, Comprehensive Stroke Center; IPTW, Inverse probability of treatment weighting; MET, metformin; MGH, Massachusetts General Hospital.

Among 8943 consecutive stroke inpatients included in our institutional dataset, 7462 patients had a final diagnosis of ischemic stroke, and 2017 had a history of diabetes mellitus. Of these, we excluded 660 who were not on any antidiabetic medications at the time of admission and 54 with a missing value for one or more outcome variables. Of the remaining 1303 patients included in the final analysis, 730 (56%) were on metformin and 573 (44%) on other antidiabetic medications (Figure 1). The mean age was 68.1 years in the MET+ and 71.2 years in the MET‐ group (SMD 0.28, *p* < 0.001).

**FIGURE 2:**
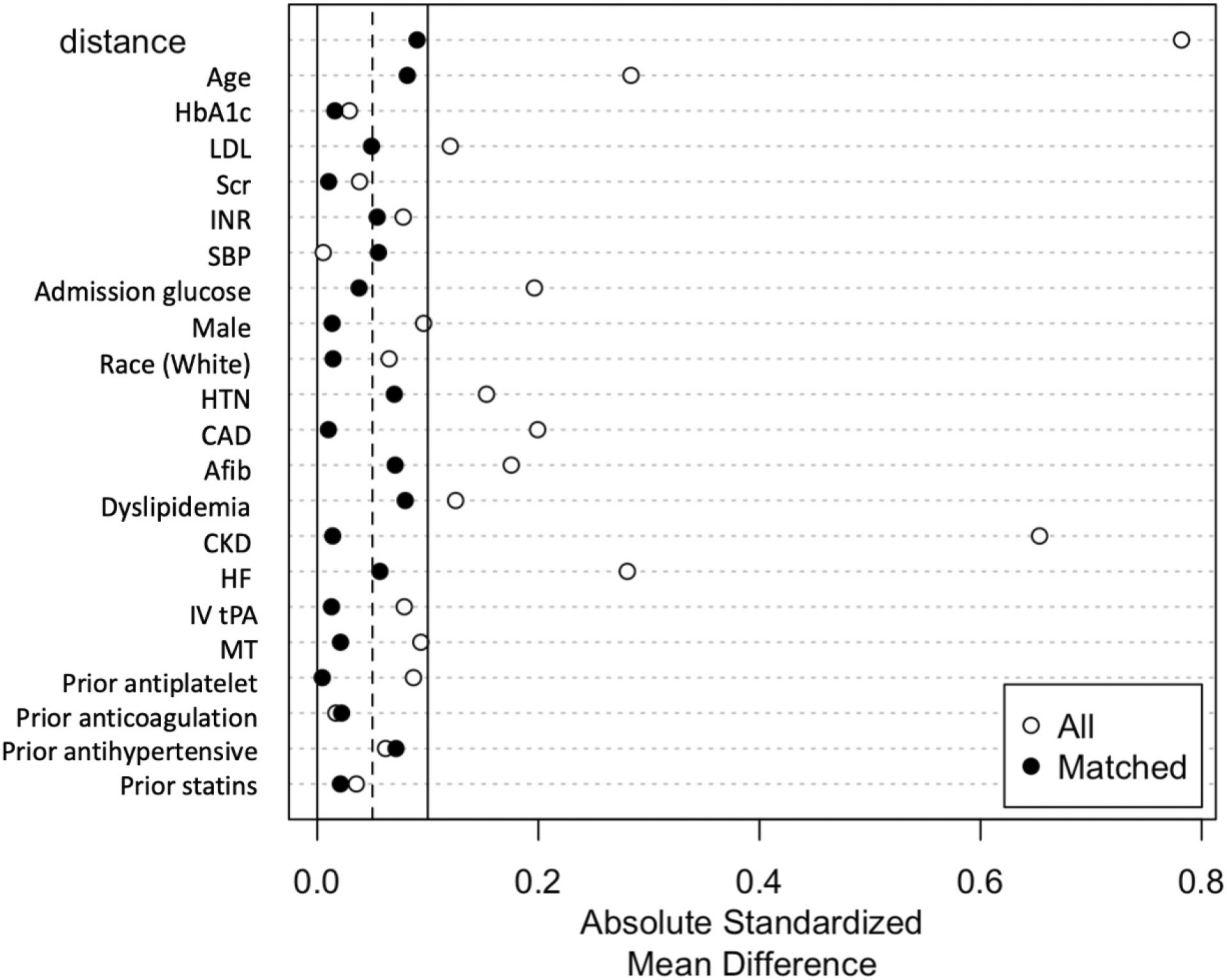
Baseline variables, assessed by standardized mean difference (SMD) before and after propensity score matching (*n* = 453 pairs). AFib, atrial fibrillation; CAD, coronary artery disease; CKD, chronic kidney disease; Cr, creatinine; HF, heart failure; HTN, hypertension; INR, international normalized ratio; IV tPA, Intravenous tissue plasminogen activator; LDL, low‐density lipoprotein; MT, mechanical thrombectomy.

CAD, CKD, and AFib were less common in the MET+ group, whereas HTN was more common. Admission glucose and serum creatinine levels were lower in the MET+ population. Baseline variables before and after PSM are shown in Table 1. We successfully matched 453 pairs of patients with MET+ and MET− status, achieving a balance across all baseline variables. The comparison of baseline characteristics between PSW− matched patients and unmatched patients is shown in Figure 2 (Appendix S1).

**TABLE 1:** Patient characteristics according to metformin treatment status before and after PSM.

Variable	At baseline				After matching		
	MET+ (*N* = 730)	MET− (*N* = 573)	*p* value	SMD	MET+ (*N* = 453)	MET− (*N* = 453)	SMD
**Demographics**							
Age, mean (SD), years	68.1 (11.6)	71.2 (12.3)	<0.001	−0.28	69.2	70.2	−0.08
Male, *n* (%)	472 (57.5)	348 (54.0)	0.19	0.10	263 (58.6)	244 (54.3)	0.09
White race, *n* (%)	559 (68.1)	457 (72.0)	0.11	−0.07	301 (67.0)	318 (70.8)	−0.08
**Past medical history**							
Hypertension, *n* (%)	726 (88.4)	533 (83.9)	0.013	0.15	385 (85.7)	383 (85.3)	0.01
Dyslipidemia, *n* (%)	545 (66.4)	391 (61.6)	0.058	0.13	282 (62.8)	276 (61.5)	−0.02
CAD, *n* (%)	213 (25.9)	221 (34.8)	<0.001	−0.20	131 (29.2)	134 (29.8)	−0.02
AFib, *n* (%)	138 (16.8)	148 (23.3)	0.002	−0.18	82 (18.3)	96 (21.4)	−0.08
Stroke, *n* (%)	228 (27.8)	186 (29.3)	0.52	−0.03	126 (28.1)	130 (29.0)	−0.02
CKD, *n* (%)	88 (10.7)	202 (31.8)	<0.001	−0.65	80 (17.8)	75 (16.7)	0.04
Heart failure, *n* (%)	80 (11.0)	113 (19.7)	<0.001	−0.28	62 (13.8)	67 (14.9)	−0.03
Smoker, *n* (%)	110 (13.4)	69 (10.9)	0.14	0.07	55 (12.2)	51 (11.4)	0.03
**Admission medications**							
Antihypertensive, *n* (%)	730 (88.9)	552 (86.9)	0.25	0.06	394 (87.8)	393 (87.5)	0.01
Antiplatelet, *n* (%)	439 (60.1)	369 (64.4)	0.12	−0.09	276 (61.5)	283 (63.0)	−0.03
Anticoagulants, *n* (%)	79 (9.6)	74 (11.7)	0.21	−0.02	42 (9.4)	42 (9.4)	0.00
Statin, *n* (%)	624 (76.0)	476 (75.0)	0.65	0.04	336 (74.8)	325 (72.4)	0.06
**Admission values**							
HbA1c, median (IQR)	7.3 (6.45–9)	7.7 (6.9–8.7)	0.03	−0.03	7.3 (6.5–8.7)	7.5 (6.6–9)	−0.02
SBP, mean (SD), mm Hg	159.2 (30.0)	159.1 (32.6)	0.97	0.01	160.0 (31.1)	158.3 (33.1)	0.06
LDL, mean (SD), mg/dL	88.2 (44.0)	82.8 (40.4)	0.12	0.025	87.7 (43.8)	85.8 (40.7)	0.04
Blood glucose, median (IQR), mg/dL	182.7 (75.2)	224.4 (145.3)	0.009	−0.18	188.3 (77.5)	193.4 (85.5)	−0.07
Serum creatinine, median (IQR), mg/dL	1.6 (1.43)	1.3 (6.4)	<0.001	−0.04	1.50 (8.2)	1.40 (1.4)	0.02
INR, mean (SD)	1.2 (0.4)	1.2 (0.3)	0.19	−0.12	1.14 (0.33)	1.14 (0.31)	<0.01
**Acute stroke therapy**							
Intravenous Thrombolysis, *n* (%)	100 (13.7)	94 (16.4)	0.17	−0.08	72 (16.0)	72 (16.0)	<0.01
MT, *n* (%)	34 (4.7)	38 (6.6)	0.12	−0.09	24 (5.3)	28 (6.2)	−0.04

*Note*: Values are presented as Mean ± Standard Deviation, number (%), or median (interquartile range). Missing values were addressed with multiple imputations.

Abbreviations: AFib, atrial fibrillation; CAD, coronary artery disease; CKD, chronic kidney disease; INR, international normalized ratio; MET, metformin; MT, mechanical thrombectomy; PSM, propensity score matching; SBP, systolic blood pressure; SMD, standardized mean difference.

### 3.2 Outcome analysis

Table 2 shows primary and secondary outcomes before and after PSM. On unmatched comparisons, the MET+ group had a significantly lower median admission NIHSS score, a lower median discharge mRS score, and lower in‐hospital mortality. After PSM, the MET+ group still had a significantly lower median admission NIHSS (SMD ‐1.18; 95% C.I., −2.13 to −0.231), a lower median discharge mRS score (SMD −0.435, 95% C.I., −0.64 to −0.23), and lower in‐hospital mortality (33 ± 7% vs. 54 ± 12%, *p* = 0.018). Table 2 additionally shows a comparison of the median values for NIHSS and mRS scores. The MET+ group had a higher proportion of patients achieving functional independence (mRS score 0–2) by discharge (34.1% vs. 22.5%, <0.001; Odds Ratio 1.09, 95% CI 1.03–1.16), and lower in‐hospital mortality (7.3% vs. 12.0%, *p* = 0.018; Odds Ratio 0.96, 95% CI, 0.93–0.99). The mean of the stabilized weights was 1.08 (SMD = 0.06). This difference remained statistically significant in the IPTW population, where the MET+ group showed greater functional independence (Odds ratio 1.09, 95% CI, 1.03–1.15) and less in‐hospital mortality (Odds Ratio 0.96, 95% CI, 0.93–0.99).

**TABLE 2:** Comparison of outcomes based on metformin treatment status, before and after propensity score matching.

Outcome	Before PSM			After PSM		
	MET+ (*N* = 730)	MET− (*N* = 573)	*p*	MET+ (*N* = 453)	MET− (*N* = 453)	*p*
Admission NIHSS	4 (1–8)	5 (2–12)	<0.001	3 (1–8)	4 (2–11)	0.011
Discharge mRS	3 (2–4)	4 (3–4)	<0.001	3 (2–4)	4 (3–4)	<0.001
In‐hospital death	47 ± 6%	69 ± 12%	<0.001	33 ± 7%	54 ± 12%	0.018

*Note*: The NIHSS and mRS score data are presented as median (interquartile range).

Abbreviations: MET−, not metformin‐treated; MET+, metformin‐treated; mRS, modified Rankin Scale score; NIHSS, National Institutes of Health Stroke Scale; PSM, propensity score matching.

### 3.3 Subgroup analysis

**FIGURE 3:**
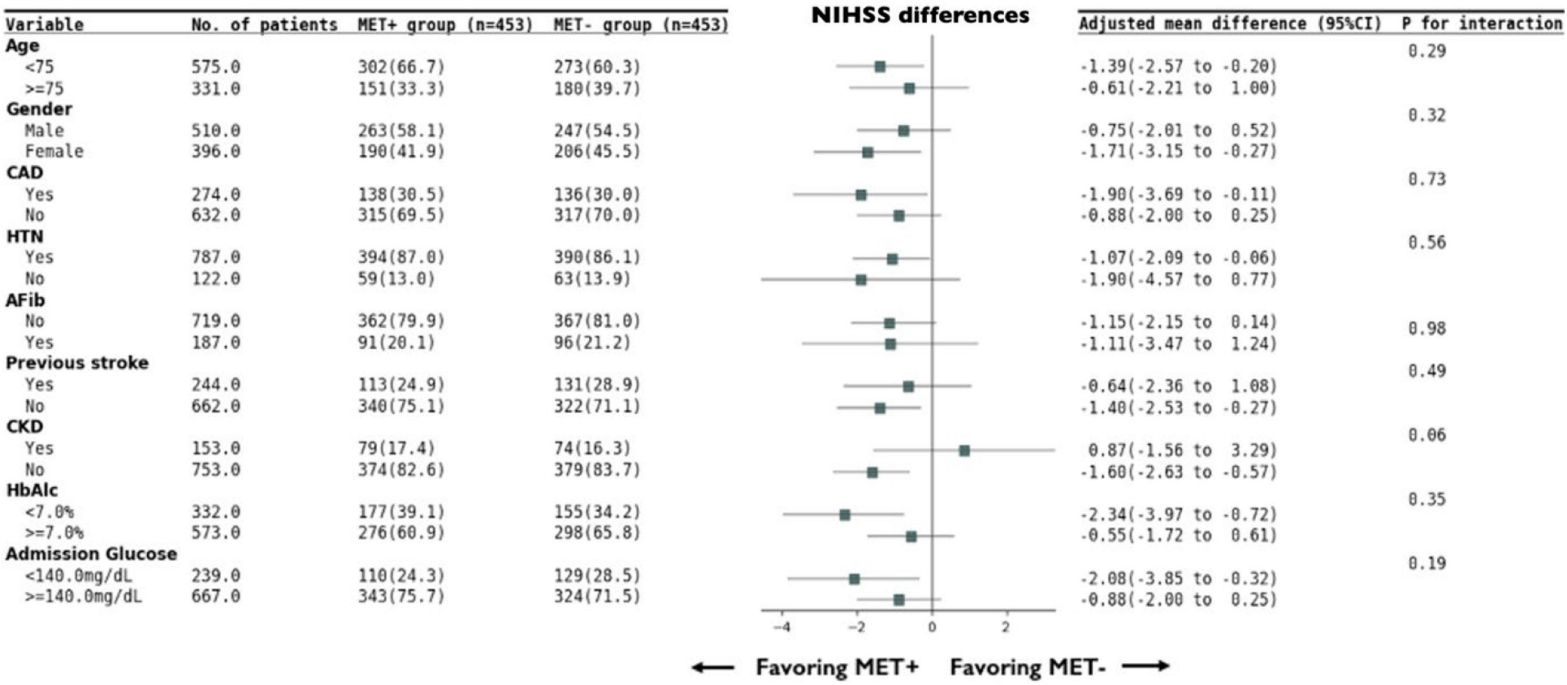
Admission NIHSS according to metformin treatment status after propensity score matching in key subgroups. AFib, atrial fibrillation; CAD, coronary artery disease; CI, confidence interval; CKD, chronic kidney disease; HTN, hypertension; MET−, not metformin‐treated; MET+, metformin‐treated.

To further investigate the potential clinical benefit of metformin on stroke severity, the adjusted mean difference of the admission NIHSS scores between the MET+ and MET− groups was calculated after PSM in key subgroups (Figure 3, S1). The propensity score was first calculated with all parameters in Table 1; subsequent analysis was completed based on the stratification by key subgroups, including age, gender, CAD, HTN, AFib, previous stroke, CKD, admission glucose level, and HbA1c value. There were no statistically significant differences in the treatment effect (Figure 3, S1).

### 3.4 Metformin dose effects

We conducted an exploratory analysis of the daily dosage of metformin at the time of stroke admission. However, no statistical significance was observed in these findings.

## 4 DISCUSSION

This retrospective analysis of our stroke dataset found that diabetics being treated with metformin had milder clinical stroke severity, less in‐hospital mortality, and better functional outcomes compared to those who were treated with antidiabetic medications other than metformin at the time of their ischemic stroke. These findings remained consistent after adjusting for confounding factors using either PSM or IIPTW analysis.

Several studies have indicated the neuroprotective effects of metformin in stroke patients.^4^, ^5^, ^6^, ^7^ Westphal et al. evaluated stroke patients post‐thrombolysis and showed that metformin pretreatment had less severe strokes, suggesting a neuroprotective effect of metformin.^5^ Naveed et al. found that metformin‐treated patients had superior outcomes in 90‐day mRS and mortality.^4^ Metformin use in stroke patients with type 2 diabetes was reported to have lower 12‐month fatality and disability by Tu et al.^6^ Paridari et al. conducted a systematic review and meta‐analysis and suggested that metformin monotherapy was effective in reducing stroke risk, while combined administration of metformin with other antidiabetic medications had no significant effect on stroke reduction.^7^ However, these studies have limitations, such as focusing on specific patient populations or having difficulty controlling the confounding factors, making it difficult to determine the true effect of metformin on stroke severity. To estimate the treatment effect and minimize the confounding bias in the general stroke population, we completed a careful and reasonable‐sized PSM study. We further confirmed the results with IPTW analysis. In our study, the beneficial effects of metformin exposure, even with the combination of other antidiabetic medications, were consistent across all analyses (PSM and IPTW analysis, analysis of dose). No statistically significant heterogeneity was found when conducting a further analysis based on the key subgroups' stratification.

Our study has revealed that pre‐treatment with metformin is associated with reduced stroke severity in patients, as well as lower in‐hospital mortality and improved functional independence upon discharge. This study expands the research population to encompass the general stroke population. Notably, we employed PSM over logistic regression for several reasons: (1) PSM can reduce confounding bias by creating comparable groups by matching based on the propensity score, while logistic regression can be susceptible to bias when a confounder effect influencing both the exposure and the outcome. (2) Ease of interpretation. PSM results in well‐balanced groups between treated and control subjects on all the covariates used for matching. (3) PSM can create more homogenous groups within the treated and control groups, leading to more precise estimates of the average treatment effect.

Metformin is known to inhibit hepatic gluconeogenesis to exert antidiabetic effects. One possible explanation of the mechanism is that chronic activation of AMPK, a protein highly expressed in neurons and activated during cerebral ischemia, may be involved.^1^, ^8^ Metformin can also increase cellular lactate levels, which can create a sublethal metabolic stress and preconditioning effect that helps prevent more damaging hypoxic ischemic injury during a stroke. Second, molecular explanations of reducing oxidative stress, apoptosis, and protein synthesis were proposed through the AMPK‐dependent pathway and the AMPK‐independent pathway.^8^ Additionally, metformin has beneficial effects on several risk factors of stroke, such as CAD, heart failure, obesity, and renal disease, which can be another explanation for this neuroprotective role.^1^, ^9^, ^10^, ^11^

Our study has several strengths. By using PSM, our research design minimizes confounding bias and ensures our groups are comparable. This allowed us to obtain more accurate estimates of the overall treatment effect. Another strength is that our study has a large sample size of 1303 ischemic stroke patients with diabetes who were taking antidiabetic medication. Third, we chose the patients who were on metformin along with other medications instead of metformin monotherapy as the treatment cohort to better reflect real‐world treatment.

We acknowledge several limitations. First, as a single‐center study in a large academic tertiary referral center in the Northeast USA, there may be race and risk factor biases and limited generalizability. Second, despite best efforts using PSM and IPTW analyses, unmeasured factors such as the effect of other antidiabetic medications may yet affect these results.^12^ Furthermore, we did not include data on stroke volume or long‐term outcomes, limiting our ability to fully understand mechanisms and investigate the potential durability of metformin's effects.

In conclusion, our results supported the clinical evidence that metformin has a stroke neuroprotective effect in patients with diabetes mellitus. Our study supports the use of metformin, regardless of the combination for additional antidiabetic medications, to treat diabetes in stroke patients. Further studies are needed to better understand mechanisms.

## FUNDING INFORMATION

None.

## CONFLICT OF INTEREST STATEMENT

Dr. Schwamm has served as a scientific consultant regarding trial design and conduct to Genentech for late window thrombolysis and as a member of the steering committee (TIMELESS NCT03785678); as a consultant on user interface design and usability to LifeImage; as a member of a Data Safety Monitoring Board (DSMB) for Penumbra (MIND NCT03342664); as the National PI for stroke prevention in AF for Medtronic (Stroke AF NCT02700945). Dr. Singhal is supported by awards from NIH‐NINDS (U24NS107243 and U10‐NS086729), the Massachusetts General Hospital Fireman Vascular Center, and the CRICO‐Risk Management Foundation. Dr. Singhal has received honoraria and royalty payments for chapter contributions to Medlink and UptoDate, Inc. He has served as a medicolegal expert witness. Dr. Singhal's wife is an employee of Biogen. The other authors have no disclosures to report.

## Supporting information


Appendix S1.

## Data Availability

The data that support the findings of this study are available from the corresponding author upon reasonable request.
