# Supplementary material for: Effectiveness of metformin pretreatment for stroke severity: A propensity score matching study
Source: CNS Neurosci Ther. 2024 Aug 21;30(8):e70004. doi: 10.1111/cns.70004 (PMC11339120; doi:10.1111/cns.70004)

**Original figures**

**Original Figure 2.** Baseline variables, assessed by standardized mean difference (SMD) before and after propensity score matching (n=453 pairs).


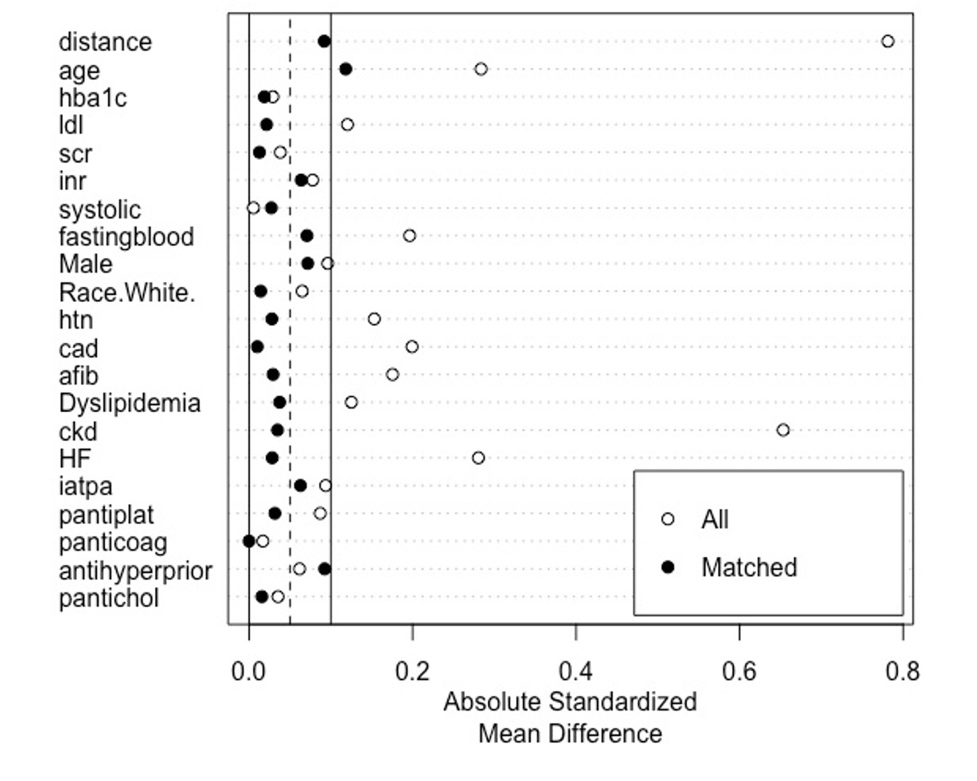


**Original Figure 3.** Admission NIHSS according to metformin treatment status after propensity score matching in key subgroups.


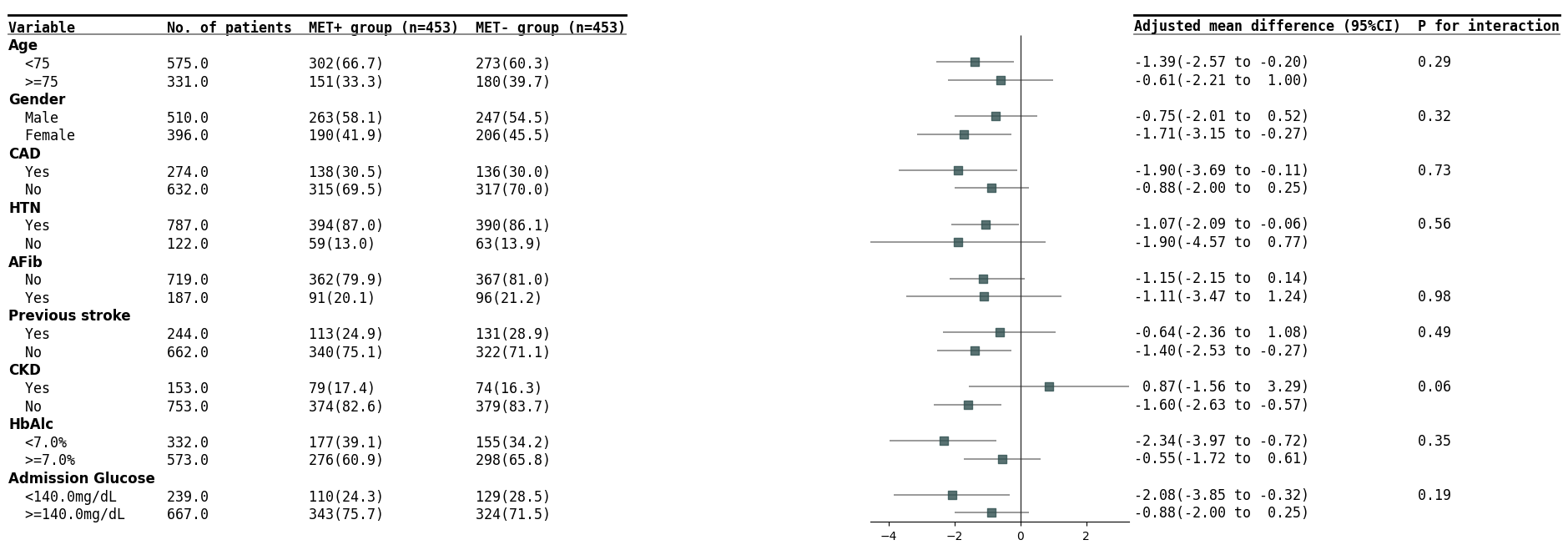

Supplement: Supplementary file 1 — Appendix S1. [file CNS-30-e70004-s001.docx]
